# Higher Bone Turnover Is Related to Spinal Radiographic Damage and Low Bone Mineral Density in Ankylosing Spondylitis Patients with Active Disease: A Cross-Sectional Analysis

**DOI:** 10.1371/journal.pone.0099685

**Published:** 2014-06-11

**Authors:** Suzanne Arends, Anneke Spoorenberg, Monique Efde, Reinhard Bos, Martha K. Leijsma, Hendrika Bootsma, Nic J. G. M. Veeger, Elisabeth Brouwer, Eveline van der Veer

**Affiliations:** 1 Rheumatology and Clinical Immunology, University of Groningen, University Medical Center Groningen, Groningen, The Netherlands; 2 Rheumatology, Medical Center Leeuwarden, Leeuwarden, The Netherlands; 3 Epidemiology, University of Groningen, University Medical Center Groningen, Groningen, The Netherlands; 4 Laboratory Medicine, University of Groningen, University Medical Center Groningen, Groningen, The Netherlands; Inserm U606 and University Paris Diderot, France

## Abstract

**Introduction:**

Ankylosing spondylitis (AS) is characterized by excessive bone formation and bone loss. Our aim was to investigate the association of bone turnover markers (BTM) with spinal radiographic damage and bone mineral density (BMD) in AS patients with active disease.

**Methods:**

201 consecutive AS outpatients of the Groningen Leeuwarden AS (GLAS) cohort were included. Serum markers of bone resorption (C-telopeptides of type-I collagen, sCTX) and bone formation (procollagen type-I N-terminal peptide, PINP; bone-specific alkaline phosphatase, BALP) were measured. Z-scores were used to correct for the normal influence that age and gender have on bone turnover. Radiographs were scored by two independent readers according to modified Stoke AS Spinal Score (mSASSS). The presence of complete bridging (ankylosis of at least two vertebrae) was considered as measure of more advanced radiographic damage. Low BMD was defined as lumbar spine and/or hip BMD Z-score ≤ −1.

**Results:**

Of the 151 patients with complete data, 52 (34%) had ≥1 complete bridge, 49 (33%) had ≥1 syndesmophyte (non-bridging), and 50 (33%) had no syndesmophytes. 66 (44%) had low BMD. Patients with bridging had significantly higher sCTX and PINP Z-scores than patients without bridging (0.43 vs. −0.55 and 0.55 vs. 0.04, respectively). Patients with low BMD had significantly higher sCTX Z-score than patients with normal BMD (−0.08 vs. −0.61). After correcting for gender, symptom duration, and CRP, sCTX Z-score remained significantly related to the presence of low BMD alone (OR: 1.60), bridging alone (OR: 1.82), and bridging in combination with low BMD (OR: 2.26).

**Conclusions:**

This cross-sectional study in AS patients with active and relatively long-standing disease demonstrated that higher serum levels of sCTX, and to a lesser extent PINP, are associated with the presence of complete bridging. sCTX was also associated with low BMD. Longitudinal studies are needed to confirm that serum levels of sCTX can serve as objective marker for bone-related outcome in AS.

## Introduction

Ankylosing spondylitis (AS) is an autoinflammatory rheumatic disease that predominantly affects the axial skeleton. The disease is characterized by the combination of inflammation, new bone formation, and bone loss. Spinal radiographic outcome related to excessive bone formation, so called osteoproliferation, comprises the formation of syndesmophytes with as final outcome complete bridging (ankylosis of two vertebrae). Eventually, complete fusion of the entire vertebral column can result in a so-called ‘bamboo spine’. [1], [2] The natural course of the disease can vary from mild to severe axial involvement and from slow to rapid radiographic progression. [1], [3] The presence of syndesmophytes at study entry is the most important predictor for the development of more extensive radiographic damage. [1], [3], [4] Furthermore, male gender, longer disease duration, smoking, human leukocyte antigen (HLA)-B27 positivity, and increased inflammatory markers were found to be related to spinal osteoproliferation.[5]–[7].

On the other hand, excessive bone loss can lead to osteopenia and osteoporosis, assessed by bone mineral density (BMD), which can already be observed at early stages of the disease. Severe vertebral bone loss may lead to vertebral fractures with increased spinal deformity. [8], [9] The presence of inflammation, low serum vitamin D levels, medication use, and decreased mobility related to pain, stiffness, and radiographic damage may contribute to bone loss in AS patients.[10]–[13].

There is a clear need for biomarkers reflecting bone-related outcome, which can help physicians in the process of decision-making on the management of AS. Previous studies in AS reported that higher serum levels of matrix metalloproteinase-3 (MMP-3), a marker of tissue remodeling, as well as lower serum levels of sclerostin and dickkopf-1, both regulators of bone turnover, were significantly associated with 2-year radiographic progression of the spine.[14]–[16] Furthermore, a relation was found between a biochemical marker of type II collagen degradation (urinary CTX-II, reflecting cartilage turnover) and increased radiographic damage or 2-year progression. [17], [18] These studies also showed a relation between a biochemical marker of type I collagen degradation (urinary CTX-I, reflecting bone resorption) and lower BMD at the hip. [17], [18] In one of our previous studies, we demonstrated that higher serum levels of CTX-I are associated with bone loss in AS patients with active disease. [11].

Bone turnover markers (BTM) may serve as objective markers for bone-related outcome in AS. A challenge of working with BTM is that serum levels change with age and there are differences for gender. Our healthy reference cohort on BTM enables us to correct BTM levels of individual AS patients for the normal influence that age and gender have on bone turnover. The aim of the present cross-sectional study was to investigate the association of BTM with spinal radiographic damage and BMD in AS patients with active disease.

## Methods

### Patients

Data collected before start of tumor necrosis factor-alpha (TNF-α) blocking therapy were used from 201 consecutive AS patients included in the Groningen Leeuwarden Ankylosing Spondylitis (GLAS) cohort [19] between November 2004 and December 2010. Patients with recent fractures or use of bisphosphonates were excluded because of their major influence on the bone metabolism. All patients were over 18 years of age, fulfilled the modified New York criteria for AS, [20] and had active disease defined by Bath AS Disease Activity Index (BASDAI) ≥4 (range 0–10) or based on expert opinion. [21].

### Ethics Statement

The study was approved by the local ethics committees of the Medical Center Leeuwarden (MCL) and University Medical Center Groningen (UMCG). All patients provided written informed consent according to the Declaration of Helsinki to participate in this study.

### BTM Assessments

Bone turnover was studied by assessment of bone resorption marker serum cross-linked telopeptide of type-I collagen (sCTX) and bone formation markers procollagen type-I N-terminal peptide (PINP) and bone-specific alkaline phosphatase (BALP). sCTX was measured by electrochemiluminescence immunoassay (ECLIA; Elecsys 2010 Roche Mannheim, Germany; inter-assay coefficient of variation (IE-CV) 10.8%), PINP by radioimmunoassay (RIA; Orion Diagnostica, Espoo, Finland; IE-CV 9.0%), and BALP by enzyme-linked immunosorbent assay (ELISA; Metra Biosystems, Mountain View, CA, USA; IE-CV 5.5%). Serum samples were stored at −20°C until analysis.

BTM Z-scores, the number of standard deviations (SD) from the normal mean corrected for age and gender, were calculated using a Dutch reference group (200 men and 350 women) checked for serum 25-hydroxyvitamin D levels >50 nmol/liter as well as for the absence of osteoporosis (BMD T-score >−2.5) after 50 years of age. Z-scores were calculated as follows: (BTM value of individual patient – mean BTM value of matched 10-year-cohort of reference group)/SD of matched reference cohort.

### Radiological Assessments

The lateral view of radiographs of the cervical and lumbar spine were blinded for patient characteristics and were scored according to the modified Stoke AS Spinal Score (mSASSS) by two trained readers (SA and AS) independent from each other. The anterior corners of lower C2 until upper T1and lower T12 until upper S1 were scored for the presence of erosions, sclerosis, and/or squaring (1 point per site), syndesmophytes (without ankylosis of vertebrae; 2 points per site), and complete bridging (ankylosis of two vertebrae; 3 points per site). The mSASSS was calculated as the sum of the scores at all individual sites (range 0–72). In case ≤3 vertebral sites were missing, these values were substituted by the mean score of the vertebrae of the same spinal segment. If >3 vertebral sites were missing, the patient was excluded from analysis (n = 6). If the mSASSS total score of both readers differed by >5 units, the X-ray was reread by the same readers. When the discrepancy of >5 units persisted following rereading, consensus was reached. [22], [23] The average mSASSS total score of both readers was used for analysis.

Inter-observer reliability for mSASSS was very good. The intraclass correlation coefficient (two-way random effects model, single measures, absolute agreement) before consensus was 0.985 (95% confidence interval (CI): 0.979–0.990). Bland-Altman analyses showed that the mean difference in mSASSS between both readers was small (0.7). The 95% limits of agreement were between −5.6 and 7.1, out of a total of 72.

The presence or absence of syndesmophytes and complete bridging was analyzed as measure of definite radiographic damage. Agreement between readers was very good, with Cohen’s kappa of 0.88 (0.80–0.96) for the presence of syndesmophytes and 0.91 (0.84–0.98) for the presence of bridging. In case of discrepancy between both readers, the consensus score was used for analysis.

### BMD Measurement

BMD at the lumbar spine (anterior-posterior projection at L1-L4) and hip (total proximal femur) were measured using DXA (Hologic QDR Discovery (UMCG) or Hologic QDR Delphi (MCL), Waltman, MA, USA). Z-scores were calculated using the NHANES reference database. Low BMD was defined as lumbar spine and/or hip BMD Z-score ≤ −1. The International Society for Clinical Densitometry recommends using BMD Z-scores instead of BMD T-scores in premenopausal women and men under the age of 50. [24].

### Clinical Assessments

Demographic data including smoking status, symptom duration, year of diagnosis, HLA-B27 status, history of extra-articular manifestations, presence of peripheral arthritis (defined as ≥1 swollen joint), and use of nonsteroidal anti-inflammatory drugs (NSAIDs) and disease-modifying antirheumatic drugs (DMARDs) were collected. Disease activity was assessed using BASDAI, [25] erythrocyte sedimentation rate (ESR), C-reactive protein (CRP), and AS disease activity score (ASDAS) calculated from BASDAI questions 2, 3, and 6, patient’s global assessment of disease activity, and CRP. [26], [27] Physical function was assessed using Bath AS Functional Index (BASFI; on a scale of 0–10). [28].

Clinical visits including biobanking and radiological and BMD measurements were performed within one year (median difference 1.6 and 0.3 months, respectively).

### Statistical Analysis

Patients with complete data available on BTM, radiology, and BMD were used in all analyses. Results were expressed as mean ± SD or median (range) for normally distributed and non-normally distributed data, respectively. Independent samples t test, Mann-Whitney U test, and Chi-Square test were used to compare differences in patient characteristics between groups.

For BTM comparisons, Kruskal-Wallis test and Mann-Whitney U test were used. Univariable logistic regression was performed to analyze the relation between BTM Z-scores and the presence or absence of complete bridging and low or normal BMD. Multivariable logistic regression was performed to correct these relations for other variables that were significantly different between these patient groups. Finally, multinomial regression was used for the combined analysis of the presence of bridging and low BMD. Patients without bridging plus normal BMD were used as reference category.

Receiver operating characteristic (ROC) analysis was performed to determine the accuracy of BTM Z-scores to discriminate between patients with or without bridging and low or normal BMD. Area under the curve (AUC) <0.70 was interpreted as poor accuracy, 0.70< AUC <0.90 as moderate accuracy, and AUC >0.90 as high accuracy. [29].

Spearman’s correlation coefficient (ρ) was used to analyze the relation between BTM Z-scores and mSASSS. P values <0.05 were considered statistically significant. Statistical analysis was performed with IBM SPSS Statistics 20 (SPSS, Chicago, IL, USA).

## Results

In total, 151 of 201 AS patients (75%) had complete data available on BTM, radiology, and BMD. Of these patients, mean age was 42.1 years (SD ±11.4), median symptom duration was 15 years (range 1–53), and 72% were male (Table 1). Patient characteristics were comparable between patients with and without complete data, except for BASFI score (5.7 vs. 6.4, p = 0.048).

**Table 1:** Characteristics of the AS study population (n = 151).

Gender (male) (n, %)	108 (72)		
Age (yrs)	42.1±11.4		
Duration of symptoms (yrs)	15 (1–53)		
Time since diagnosis (yrs)	7 (0–44)		
HLA-B27+ (n, %)	120 (82)		
History of IBD (n, %)	14 (9)		
History of uveitis (n, %)	44 (29)		
History of psoriasis (n, %)	9 (6)		
Peripheral arthritis (n, %)	29 (19)		
Current smoking (n, %)†	45 (41)		
Current NSAID use (n, %)	108 (72)		
Current DMARD use (n, %)	36 (24)		
Current steroid use (n, %)‡	11 (7)		
BASDAI (range 0–10)	6.1±1.7	BASDAI ≥4 (n, %)*	135 (89)
ASDAS_CRP_	3.8±0.8	ASDAS_CRP_≥2.1 (n, %)*	146 (99)
ESR (mm/h)	21 (2–90)		
CRP (mg/l)	13 (2–99)		
BASFI (range 0–10)	5.7 (0.4–9.7)		
sCTX Z-score	−0.34 (−2.58–5.38)		
PINP Z-score	0.23 (−1.75–8.77)		
BALP Z-score	0.32 (−2.59–10.38)		
mSASSS (range 0–72)	12 (1–72)	≥1 syndesmophyte (n, %)	101 (67)
		≥1 complete bridge (n, %)	52 (34)
LS BMD Z-score	–0.33±1.49	LS BMD Z-score ≤–1 (n, %)	52 (36)
		LS BMD Z-score ≤–2 (n, %)	16 (11)
Hip BMD Z-score	–0.23±1.06	Hip BMD Z-score ≤–1 (n, %)	38 (25)
		Hip BMD Z-score ≤–2 (n, %)	6 (4)

Values are mean ± SD or median (range) unless otherwise indicated.

† Data were available in 72% of the patients.

‡ Of these patients, 3 used systemic corticosteroids (prednison 5 mg/d n = 2, budenofalk 6 mg/d n = 1) and 8 used local corticosteroids (nose drops n = 3, eye drops n = 2, inhaled n = 2, skin cream n = 1, injection knee n = 1).

*Active disease based on BASDAI (21) or ASDAS [37].

AS, ankylosing spondylitis; HLA-B27+, human leukocyte antigen B27 positive; IBD, inflammatory bowel disease; NSAID, non-steroidal anti-inflammatory drug; DMARD, disease-modifying antirheumatic drug; BASDAI, Bath AS disease activity index; ASDAS, AS disease activity score; ESR, erythrocyte sedimentation rate; CRP, C-reactive protein; BASFI, Bath AS functional index; PINP, procollagen type I N-terminal peptide; BALP, bone-specific-alkaline phosphatase; sCTX, serum C-telopeptide of type I collagen; mSASSS, modified stoke AS spinal score; LS, lumbar spine; BMD, bone mineral density.

### Radiological Damage and BTM

**Figure 1:**
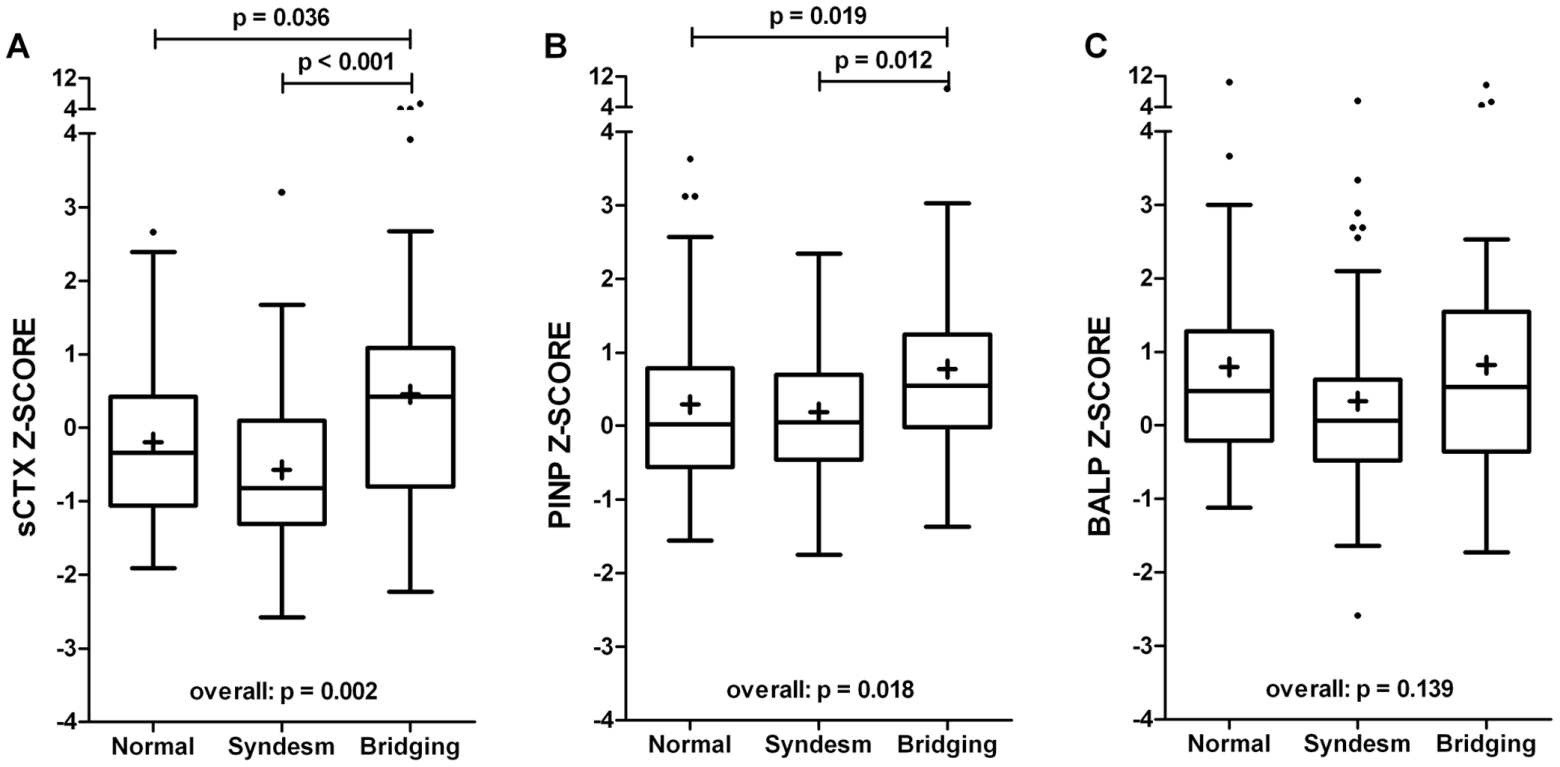
Bone turnover in AS patients with ≥1 complete bridge (n = 52), ≥1 non-bridging syndesmophyte (n = 49), and without syndesmophytes (n = 50). A) bone resorption marker sCTX, B) bone formation marker PINP, C) bone formation marker BALP. Box-and-whisker plots (Tukey): boxes indicate medians with interquartile ranges;+indicate means; whiskers indicate 1.5 times the interquartile distances; · indicate outliers.

Of the 151 patients, 52 (34%) had at least one complete bridge, 49 (33%) had at least one syndesmophyte (non-bridging), and 50 (33%) had no syndesmophytes. Patients with bridging had significantly higher sCTX and PINP Z-scores than patients with non-bridging syndesmophytes or without syndesmophytes, whereas no significant difference was found in BALP Z-score (Figure 1).

No significant differences in BTM Z-scores were found between patients with and without non-bridging syndesmophytes (Figure 1) and these two categories were combined in further analyses. The presence of complete bridging was considered as measure of more advanced radiographic damage. In these patients, the median number of bridges was 4 (range 1–12). The median difference in Z-score between patients with (n = 52) and without (n = 99) bridging was larger for sCTX than for PINP (0.98 vs. 0.51). In addition, patients with bridging were significantly more often male and had more long-standing disease (Table 2).

**Table 2:** Characteristics of AS patients with or without complete bridging and with low or normal BMD.

	Bridging^a^	No bridging	Low BMD^b^	Normal BMD
Number of patients	52	99	66	85
Gender (male) (n, %)	47 (90)	61 (62)*	51 (77)	57 (67)
Duration of symptoms (yrs)	24 (2–53)	11 (1–35)*	13 (2–47)	18 (1–53)†
Time since diagnosis (yrs)	14 (0–44)	3 (0–26)*	7 (0–44)	6 (0–41)
HLA-B27+ (n, %)	43 (88)	77 (79)	50 (78)	70 (84)
Current smoking (n, %)	16 (47)	29 (39)	18 (36)	27 (46)
Current NSAID use (n, %)	40 (77)	68 (69)	50 (76)	58 (68)
BASDAI (range 0–10)	5.9±1.7	6.1±1.6	5.9±1.8	6.2±1.6
ASDAS_CRP_	3.8±0.8	3.7±0.8	3.8±0.9	3.7±0.7
ESR (mm/h)	20 (3–76)	21 (2–90)	23 (2–90)	17 (2–76)
CRP (mg/l)	14 (2–99)	13 (2–70)	16 (2–99)	10 (2–64)†
BASFI (range 0–10)	6.2 (1.1–9.7)	5.6 (0.4–9.3)	5.5 (0.4–9.7)	5.7 (0.5–9.3)
sCTX Z-score	0.43 (−2.23–5.38)	−0.55 (−2.58–3.20)*	−0.08 (−1.89–4.01)	−0.61 (−2.58–5.38)†
sCTX (pg/ml)	239.4 (31.0–618.7)	175.8 (13.4–657.1)*	227.6 (35.6–657.1)	160.3 (13.4–618.7)†
PINP Z-score	0.55 (−1.37–8.77)	0.04 (−1.75–3.63)*	0.12 (−1.56–3.63)	0.28 (−1.75–8.77)
PINP (µg/l)	47.6 (17.9–132.5)	41.6 (16.0–101.5)	50.2 (16.4–101.5)	41.8 (16.0–132.5)
BALP Z-score	0.52 (−1.73–9.68)	0.28 (−2.59–10.38)	0.17 (−1.64–10.38)	0.43 (−2.59–9.68)
BALP (U/L)	18.2 (6.9–67.2)	17.6 (1.6–41.2)	18.3 (8.9–41.2)	17.5 (1.6–67.2)
mSASSS (range 0–72)	40 (11–72)	7 (1–29)*	10 (1–72)	15 (2–72)†
LS BMD Z-score	0.09±1.59	−0.52±1.40*	−1.47±0.93	0.60±1.18†
Hip BMD Z-score	−0.25±0.92	−0.21±1.13	−0.97±0.76	0.35±0.89†

Values are mean ± SD or median (range) unless otherwise indicated.

a Defined as ankylosis of at least two vertebrae.

b Defined as lumbar spine and/or hip BMD Z-score ≤ −1.

*p<0.05 compared to patients with at least one complete bridge.

† p<0.05 compared to patients with low BMD.

See Table 1 for abbreviations.

No significant differences in extra-articular manifestations or peripheral arthritis were found between patients with or without complete bridging and low or normal BMD (data not shown).

In univariable logistic regression, sCTX Z-score (odds ratio (OR): 1.62, 95% CI: 1.22–2.14) and PINP Z-score (OR: 1.43, 1.06–1.93) were significantly associated with the presence of complete bridging. The amount of variance explained by sCTX and PINP was 11.6% and 5.5%, respectively. ROC analysis showed that the AUC discriminating between patients with and without bridging was 0.66 (0.57–0.75) for sCTX Z-score and 0.64 (0.55–0.73) for PINP Z-score. After correcting for gender and symptom duration, sCTX Z-score (OR: 1.50, 1.09–2.07) remained significantly related to the presence of bridging, while PINP Z-score almost reached significance (OR 1.48, 0.99–2.22). Correcting for time since diagnosis instead of symptom duration yielded similar results (sCTX: OR 1.57, 1.16–2.13; PINP: OR 1.62, 1.12–2.35).

The mSASSS correlated significantly with sCTX Z-score (ρ = 0.200, p = 0.016) and PINP Z-score (ρ = 0.180, p = 0.030); no relation was found with BALP Z-score (ρ = 0.010, p = 0.910).

### Bone Mineral Density and BTM

**Figure 2:**
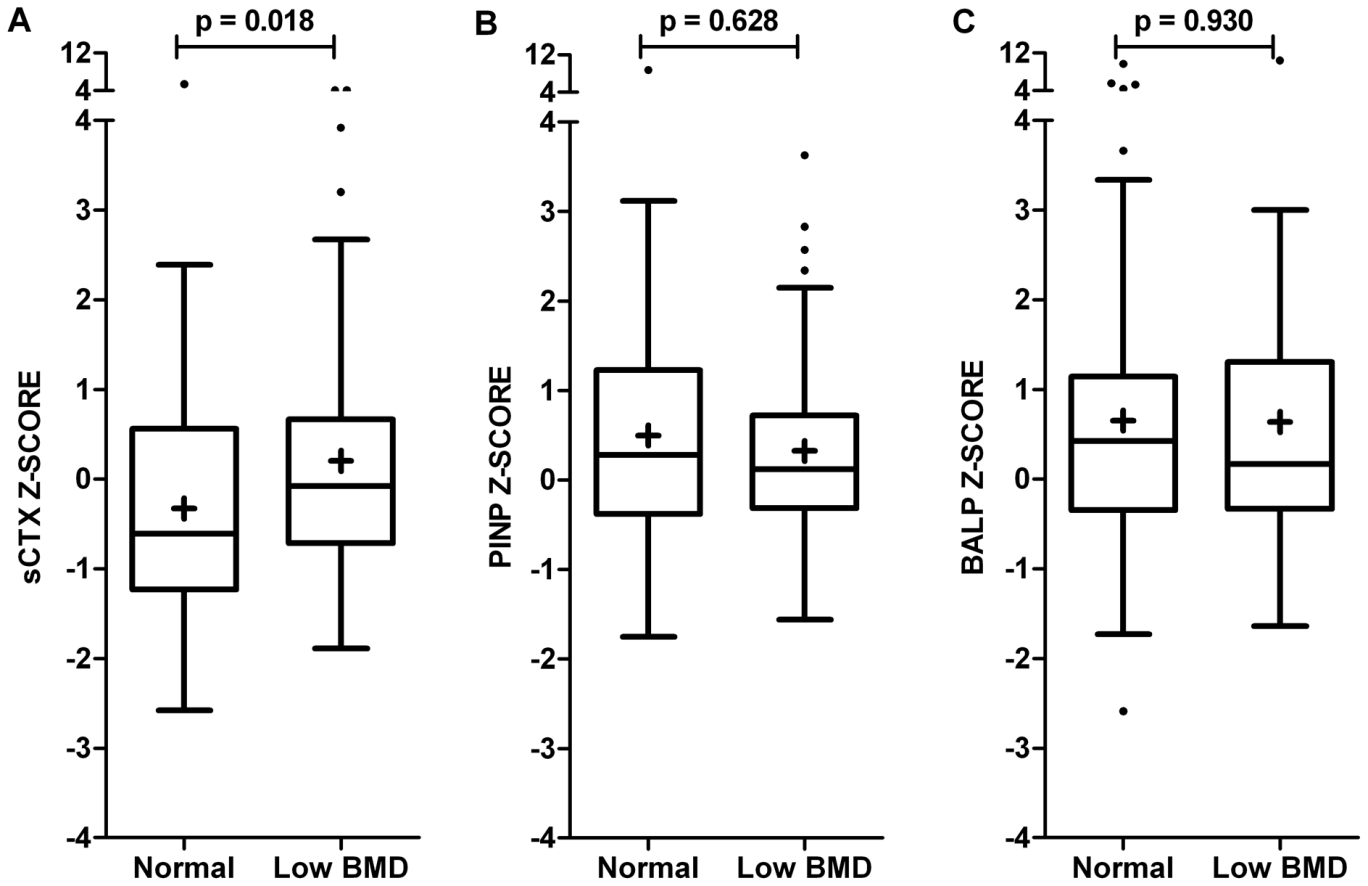
Bone turnover in AS patients with low (n = 66) and normal (n = 85) BMD. A) bone resorption marker sCTX, B) bone formation marker PINP, C) bone formation marker BALP. Box-and-whisker plots (Tukey): boxes indicate medians with interquartile ranges;+indicate means; whiskers indicate 1.5 times the interquartile distances; · indicate outliers.

Of the 151 patients, 66 (44%) had lumbar spine and/or hip BMD Z-score ≤ −1 and 18 (12%) had BMD Z-score ≤ −2. Patients with low BMD (n = 66) had significantly higher sCTX Z-scores than patients with normal BMD (median difference 0.53). No significant difference was found in PINP and BALP Z-scores (Figure 2). In addition, patients with low BMD had significantly higher CRP levels and shorter disease duration (Table 2).

In univariable logistic regression, sCTX Z-score (OR: 1.35, 1.05–1.74) was significantly associated with the presence of low BMD. The amount of variance explained by sCTX Z-score was 5.1%. The AUC of sCTX Z-score was 0.61 (0.52–0.70) to discriminate between patients with low and normal BMD. This relation remained significant (OR: 1.38, 1.04–1.83) after correcting for CRP and symptom duration.

### Combined Analysis

**Figure 3:**
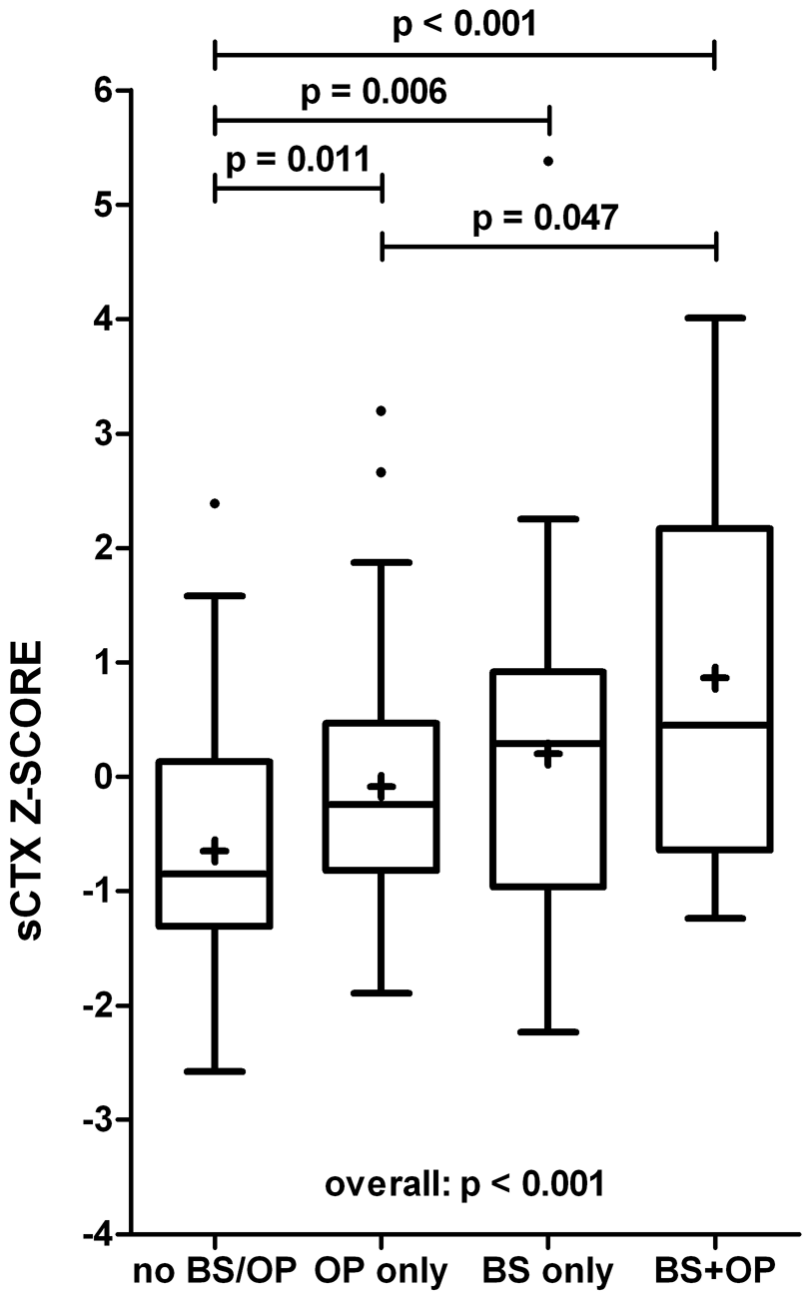
Bone resorption marker sCTX in AS patients with complete bridging, low BMD or both. Box-and-whisker plots (Tukey): boxes indicate medians with interquartile ranges;+indicate means; whiskers indicate 1.5 times the interquartile distances; · indicate outliers. BS: complete bridging, OP: low BMD.

Both complete bridging and low BMD were present in 20 of 151 (13%) patients. 32 (21%) had only bridging, 46 (31%) had only low BMD, and 53 (35%) did not have bridging or low BMD. sCTX Z-score was significantly higher in patients with low BMD, bridging, or both compared to patients without bridging and normal BMD. Furthermore, patients with both bridging and low BMD had significantly higher sCTX Z-scores than patients with only low BMD (Figure 3).

In univariable multinomial logistic regression, sCTX Z-score was significantly associated with the presence of low BMD alone (OR: 1.59), bridging alone (OR: 1.88), and bridging in combination with low BMD (OR: 2.51). The amount of variance explained by sCTX Z-score was 14.4%. The AUC of sCTX Z-score was 0.78 (0.67–0.89) to discriminate between patients with bridging plus low BMD and patients without bridging plus normal BMD.

After correcting for gender, symptom duration, and CRP, sCTX Z-score remained significantly related to the presence of low BMD alone (OR: 1.60), bridging alone (OR: 1.82), and bridging in combination with low BMD (OR: 2.26) (Table 3). The total amount of variance explained by the full model was 45.9%. Correcting for BASDAI, ASDAS, or ESR instead of CRP yielded similar results (data not shown).

**Table 3:** Multinomial regression analysis for the relation between bone resorption marker sCTX and the presence of complete bridging and/or low BMD.

	Low BMD		Bridging		Low BMD and bridging	
	OR (95% CI)	p-value	OR (95% CI)	p-value	OR (95% CI)	p-value
sCTX Z-score	1.59 (1.90–2.31)	0.016	1.88 (1.26–2.81)	0.002	2.51 (1.61–3.90)	<0.001
sCTX Z-score (corrected model)‡	1.60 (1.04–2.45)	0.032	1.82 (1.12–2.94)	0.015	2.26 (1.37–3.73)	0.002

Patients without bridging and normal BMD were used as reference category.

‡ Corrected for gender, symptom duration, and CRP.

See Tables 1 and 2 for abbreviations and definitions, respectively.

## Discussion

The present cross-sectional study within the GLAS cohort showed that higher serum levels of bone resorption marker sCTX and bone formation marker PINP were significantly associated with the presence of complete bridging in AS patients with active disease. The relation of sCTX with bridging remained statistically significant after correcting for gender and disease duration. Our finding that patients with bridging were more often male and had longer disease duration is in accordance with earlier findings in AS. [4], [30], [31].

In clinical studies concerning the monitoring of osteoporosis treatment and fracture risk prediction, the use of sCTX and PINP, both products of type I collagen, has been recommended. [32] Neither sCTX nor PINP are specific to bone, but the highest contributions are probably bone derived. BALP, an enzyme that has a central role in the mineralization process after osteoid forming, is specific for bone, but has some cross-reactivity with liver isoform (up to 20%). Although PINP and BALP show smaller circadian rhythm, sCTX was closer related to osteoproliferation than PINP; the median difference in Z-score between AS patients with and without bridging was 0.98 and 0.51, respectively. Surprisingly, no significant association was found for BALP.

Previous studies searching for biomarkers that reflect structural damage in AS have shown that higher serum levels of MMP-3 significantly predicted 2-year radiographic progression in multivariable analysis. [14] Furthermore, lower serum levels of Wnt signalling pathway inhibitors sclerostin and dickkopf-1 were significantly associated with 2-year radiographic progression in univariable analysis. [15], [16] Another candidate, cartilage turnover marker urinary cross-linked telopeptide of type-II collagen (uCTX-II) was found to be independently related to radiological damage at baseline and 2-year radiological progression. However, the amount of variance explained by uCTX-II was small (full model R^2^ was 6%). [18] In contrast to our findings, Vosse et al. could not demonstrate a relation between uCTX-I and structural damage. [18] The most likely explanation for this difference is that measuring CTX in urine has more variation compared to serum as well as that sCTX levels were not corrected for age and gender in the former study.

Besides osteoproliferation, AS is characterized by bone loss, which can lead to a reduction in BMD and/or vertebral fractures. In the present study, a significant association between sCTX and low BMD was found. Analyzing lumbar spine and hip BMD separately, we found only a significant difference in sCTX for low hip BMD (data not shown). The relation between CTX-I and low hip BMD has been reported before. [17], [18] Serum levels of PINP and BALP were comparable between patients with normal and low BMD. These findings are in accordance with two previous studies, which also found no significant correlation between PINP or BALP and BMD in small groups of AS patients. [33], [34].

Finally, we analyzed the combination of excessive bone formation (vertebral ankylosis) and bone loss (low BMD). sCTX Z-score was significantly higher in AS patients with low BMD, complete bridging, or both compared to patients without bridging and normal BMD. Interestingly, these differences remained significant after correcting for gender, disease duration, and CRP, suggesting that serum levels of sCTX reflect bone-related outcome in AS. The accuracy of sCTX Z-score to discriminate between patients with bridging plus low BMD and patients without bridging plus normal BMD was moderate (AUC of 0.78).

A striking finding was that AS patients without bridging and normal BMD had a median sCTX Z-score of −0.85. Further exploration of the reduction in this bone resorption marker in this group of AS patients is needed.

With pre-analytical standardization of blood sampling as well as standardization of assays, [32] sCTX and PINP meet most of the requirements of the Outcome Measures in Rheumatology (OMERACT) validation criteria for soluble biomarkers reflecting structural damage. [35] Regardless the variation in BTM levels between individual patients, the present cross-sectional study shows that serum levels of sCTX and PINP are higher in AS patients with bridging. Furthermore, both BTM correlate significantly with mSASSS, the structural damage endpoint for AS. These rather weak correlations can probably be explained by the fact that the presence of erosions, sclerosis, and/or squaring can already result in mSASSS scores up to 24, without evidence of bony proliferation on radiographs. To exclude the influence of these relatively minor radiographic changes, we used the presence of complete bridging as measure of more advanced radiographic damage in AS. Interestingly, BTM were found to be associated with the presence of complete bridging, but not with the presence of syndesmophytes. A possible explanation may be that syndesmophytes can vary in size and severity. Complete bridging is considered as the final outcome (ankylosing of two vertebrae).

Although very interesting, our results should be interpreted with some caution due to the cross-sectional design and only AS patients with active and relatively long-standing disease were analyzed. To confirm our results, further studies should include also patients without active disease as well as patients in early stages of the disease. TNF-α blocking agents have shown to be very effective in controlling systemic inflammation and improving clinical outcome in AS. [2] In addition, we found a significant decrease in sCTX after starting TNF-α blocking therapy. [19] In the present analysis, disease activity did not influence the association between sCTX Z-score and the presence of complete bridging or low BMD. However, BTM and inflammatory markers in serum are systemic measurements and the relation with local inflammatory processes was not investigated.

In 50 patients (25%), part of the data on BTM (n = 10), radiology (n = 38), and BMD (n = 15) was missing due to logistic reasons and they were excluded from all analyses. Patient characteristics were comparable between patients with and without complete data, except for somewhat higher BASFI score in patients without complete data. When including all available data per analysis, similar results were found as in the complete case analysis. Similar results were also observed after excluding patients who used corticosteroids (data not shown).

Patients with complete bridging were identified to have significantly longer disease duration and higher lumbar spine BMD compared to patients without bridging. Hip BMD was similar between both groups. On the other hand, patients with low lumbar spine BMD had significantly shorter disease duration than patients with normal BMD. These findings suggest that bridging causes an overestimation in lumbar spine BMD measured by DXA in patients with advanced AS. This is in line with our previous finding that the difference between lumbar spine and hip BMD correlated positively with disease duration. [11] Furthermore, Karberg et al. showed that in AS patients with short disease duration (<5 years), low BMD was found more frequently in the spine, whereas in patients with longer disease duration (>10 years), osteoporosis was more frequent found in the hip. [36] For this reason, we used both lumbar spine and hip to define low BMD in our study. However, since we analyzed a group of AS patients with relatively long-standing disease (median symptom duration of 15 years), the percentage of patients with low BMD may be underestimated. BMD Z-scores were used because of the young age (mean 42 years) of the AS population [24].

The prevalence of low BMD found in our cohort was high compared to the general population. Of the AS patients, 44% had lumbar spine and/or hip BMD Z-score ≤ −1 and 12% had BMD Z-score ≤ −2. Due to the small number of patients with BMD Z-score ≤ −2, separate analyses using this threshold could not be performed, which may limit the clinical interpretation of our results. Patients who used bisphosphonates (because of severe osteoporosis) were excluded because of the large reduction in BTM.

## Conclusion

This cross-sectional analysis in AS patients with active disease demonstrated that higher serum levels of sCTX, and to a lesser extent PINP, are associated with the presence of complete bridging. Higher serum levels of sCTX were also associated with low BMD. Longitudinal studies are needed to confirm that BTM, especially sCTX, can serve as objective markers for bone-related outcome as well as to investigate whether BTM can predict spinal radiographic progression and decrease in BMD in AS.
